# Complete Genome Sequencing of a Novel Strain of Sapelovirus A Circulating in Vietnam

**DOI:** 10.1128/MRA.00959-19

**Published:** 2019-09-12

**Authors:** Hoang Vu Dang, Tomoko Kato, Tatsuya Nishi, Ha Thanh Tran, Kohtaro Miyazawa, Takehiro Kokuho

**Affiliations:** a Department of Biochemistry and Immunology, National Institute of Veterinary Research, Hanoi, Vietnam; b Division of Transboundary Animal Disease, National Institute of Animal Health, National Agriculture and Food Research Organization, Tokyo, Japan; c Division of Virology and Epidemiology, National Institute of Animal Health, National Agriculture and Food Research Organization, Ibaraki, Japan; KU Leuven

## Abstract

Sapelovirus A (SV-A) is currently spreading as an enteric pathogen of pigs worldwide. We isolated SV-A strain XTND/2018 from the small intestine of a dead pig with severe diarrhea in the north of Vietnam and determined the genomic sequence. This is the first report of the genomic sequence of SV-A circulating in Vietnam.

## ANNOUNCEMENT

Sapelovirus A (SV-A) is a nonenveloped single-stranded positive-sense RNA virus belonging to the family *Picornaviridae*, genus *Sapelovirus*. SV-A may cause acute diarrhea, respiratory and reproductive disorders, and polioencephalomyelitis in pigs, although the infection is most frequently asymptomatic (1–4). SV-A infects mainly through the oral route, replicates in the gastrointestinal epithelia, and is excreted into feces, which are a source for secondary infections (5). A recent study demonstrated that the prevalence of SA-V was 31% among pigs affected by porcine endemic diarrhea (6). Since 2018, continuous outbreaks of severe diarrhea have been found at commercial pig farms in Nam Dinh Province in the north of Vietnam. In order to identify causative agents of the disease, we attempted virus isolation from a homogenate of small intestine of a dead pig with diarrhea and recovered a novel strain of SV-A using cultures of a porcine kidney cell line (CPK). Here, we determined the whole-genome sequence of the strain XTND/2018 by next-generation sequencing. The virus was propagated in the culture of CPK cells for 4 to 5 days at 37°C until cytopathic effect was observed. Virus-containing culture supernatants were concentrated 125 times with a Vivaspin 20 ultrafiltration unit (Sartorius) and then subjected to total RNA extraction with the viral RNA extraction kit (Roche). The cDNA was synthesized with the PrimeScript double-strand cDNA synthesis kit (TaKaRa) and treated with the Ion Xpress Plus fragment library kit (Life Technologies) for library preparation. The cDNA library was sequenced with an Ion Torrent PGM sequencer (Life Technologies) using the 314v2 chip and the Ion PGM sequencing 200 kit (Life Technologies). The collected data of 29,649 reads in total (the average read length was 181 bp) were assembled *de novo* using the Torrent Suite version 5.0.5 and the SPAdes software version 3.1.0 with default parameters (Life Technologies). The assembly was 7,301 nucleotides (nt) long with an average coverage depth of 6.39×. The sequences at the 5′ and 3′ termini were amplified with the SMARTer rapid amplification of cDNA ends (RACE) 5′/3′ kit (Clontech) using the universal primer in combination with 5′ and 3′ region-specific primers, 5′-ACCAAAGGAGCACGCCGAAG-3′ and 5′-ACAGGAACAAAGTTAACAGATGTAACATTCCTGAA-3′, respectively, and determined using a SeqStudio genetic analyzer (Applied Biosystems). The final genome assembly of strain XTND/2018 was 7,495 nt in size with a 40.1% G+C content encoding a 6,972-nt polyprotein. The polypeptide coding sequence showed the highest nucleic acid sequence similarity (90%) and amino acid identity (98%) with Chinese strain HuN22 (GenBank accession number MF440650) based on NCBI BLAST analysis (analyzed 26 July 2019). To the best of our knowledge, this is the first report on SV-A of Vietnamese origin. Although the prevalence of the virus in Vietnam is currently not well understood, we speculate that SV-A may affect the health status of pigs by itself or in combination with other coexisting pathogens, such as enterovirus, coronavirus, or rotavirus, nationwide. The data we present here will be valuable for further endemicity and epidemiological investigations of SV-A and for providing a better understanding of this organism’s role in economic loss due to gastroenteritis in pigs in Vietnam.

### Data availability.

The genome sequence of the XTND/2018 strain has been deposited in DDBJ/ENA/GenBank (accession number LC493088). The raw sequence reads were deposited in the DDBJ Sequence Read Archive (DRA) under the BioProject and DRA accession numbers PRJDB8629 and DRA008757, respectively.
